# Lineage-specific canonical and non-canonical activity of EZH2 in advanced prostate cancer subtypes

**DOI:** 10.21203/rs.3.rs-3935288/v2

**Published:** 2024-03-04

**Authors:** Varadha Balaji Venkadakrishnan, Adam G. Presser, Richa Singh, Matthew A. Booker, Nicole A. Traphagen, Kenny Weng, Nathaniel C. Voss, Navin R. Mahadevan, Kei Mizuno, Loredana Puca, Osasenaga Idahor, Sheng-Yu Ku, Martin K. Bakht, Ashir A. Borah, Zachary T. Herbert, Michael Y. Tolstorukov, David A. Barbie, David S. Rickman, Myles Brown, Himisha Beltran

**Affiliations:** Dana-Farber Cancer Institute; Dana-Farber Cancer Institute; Weill Cornell Medicine; Dana-Farber Cancer Institute; Dana-Farber Cancer Institute; Dana-Farber Cancer Institute; Dana-Farber Cancer Institute; Dana-Farber Cancer Institute; Dana Farber Cancer Institute; Division of Medical Oncology, Weill Cornell Medicine; Dana-Farber Cancer Institute; Dana-Farber Cancer Institute; Dana-Farber Cancer Institute; Broad Institute of Harvard and MIT; Dana-Farber Cancer Institute; Dana-Farber Cancer Institute; Dana-Farber Cancer Institute; Weill Cornell Medicine; Dana-Farber Cancer Institute; Dana-Farber Cancer Institute

## Abstract

Enhancer of zeste homolog 2 (EZH2) is a histone methyltransferase and emerging therapeutic target that is overexpressed in most castration-resistant prostate cancers and implicated as a driver of disease progression and resistance to hormonal therapies. Here we define the lineage-specific action and differential activity of EZH2 in both prostate adenocarcinoma (PRAD) and neuroendocrine prostate cancer (NEPC) subtypes of advanced prostate cancer to better understand the role of EZH2 in modulating differentiation, lineage plasticity, and to identify mediators of response and resistance to EZH2 inhibitor therapy. Mechanistically, EZH2 modulates bivalent genes that results in upregulation of NEPC-associated transcriptional drivers (e.g., ASCL1) and neuronal gene programs, and leads to forward differentiation after targeting EZH2 in NEPC. Subtype-specific downstream effects of EZH2 inhibition on cell cycle genes support the potential rationale for co-targeting cyclin/CDK to overcome resistance to EZH2 inhibition.

## Introduction

Lineage plasticity is a mechanism of treatment resistance seen across many cancer types (1). In prostate cancer, lineage plasticity is associated with resistance to hormonal therapies which can manifest clinically as histologic transformation from prostate adenocarcinoma (PRAD) to neuroendocrine prostate cancer (NEPC) (2). Lineage plasticity occurs in up to 15–20% of late-stage prostate cancers and is associated with poor prognosis (3, 4). Expression of androgen receptor (AR), the main driver of PRAD, is often lost in NEPC along with other luminal prostate features (5). Epigenetic dysregulation is a major driver of cancer lineage plasticity (6, 7). We reported that NEPC arises clonally from PRAD with shared genomic features (8), acquiring distinct changes in DNA methylation and chromatin accessibility that associate with dysregulation of lineage-determining transcription factors that distinguish NEPC from PRAD (8–11). Epigenetic de-repression of cell fate commitment transcription factors likely facilitates lineage plasticity and NEPC progression (12). The polycomb repressive complex (PRC2) regulates the epigenetic mark H3K27me3 associated with transcriptional repression (13), and this function is mediated by its histone methyltransferase component EZH2. EZH2 is overexpressed in most castration-resistant prostate cancers including in NEPC and has emerged as a therapeutic target (8, 14).

The PRC2 complex plays an important role during cell fate transitions and differentiation during normal development (15). In this context, PRC2 acts to silence lineage-specific transcription factors (16), and loss of PRC2 in mouse models specifically induces bivalent promoters bearing both active H3K4me3 and repressive H3K27me3 acquired upon attaining a differentiated state (17, 18). In addition to its PRC2-related canonical function associated with cell identity, there is also a PRC2-independent, transcriptional co-activator function of EZH2 (19). In prostate adenocarcinoma, this non-canonical function of EZH2 can collaborate with the AR transcriptional machinery to drive AR-signaling (19–21) or with N-myc to modulate plasticity (22, 23). The efficacy of EZH2 inhibition in PRAD preclinical models is attributable in part to the blockade of non-canonical EZH2 target genes (19). The molecular relevance of the EZH2 co-activator function in the absence of AR, as seen in NEPC, requires further investigation.

In this study, we sought to define the mechanism of action of EZH2 in both PRAD and NEPC to determine how the underlying lineage state can impact EZH2 function and response to EZH2 inhibition in prostate cancer. We highlight how differential PRC2-mediated canonical activity contributes to a lack of lineage reversal in NEPC upon EZH2 inhibition, and in fact leads to forward differentiation towards a more terminally differentiated NE state. We investigate the relevance of the non-canonical EZH2 function which pointed to cell cycle mediators of response to EZH2 inhibition and a potential opportunity to co-target the cyclin/CDK pathway to overcome resistance.

## Results

### NEPC preclinical models show modest response to EZH2 inhibitor therapy

**Figure 1:**
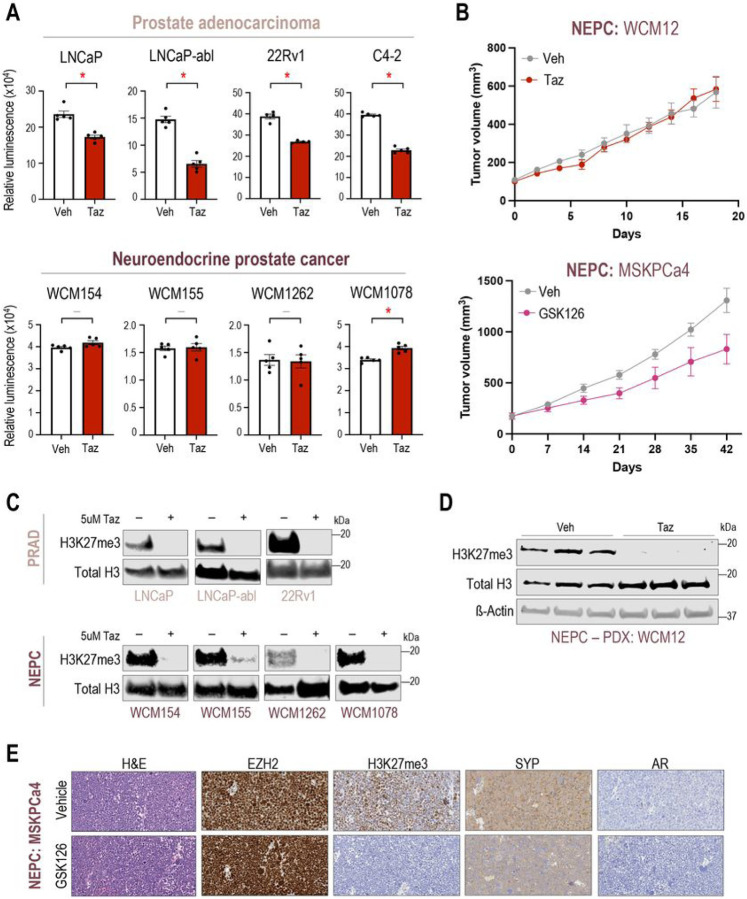
NEPC preclinical modes show modest response to EZH2i **(A)** Prostate cancer models (Top panel – AR-driven PRAD; bottom panel – AR-indifferent NEPC) were treated with vehicle (Veh; DMSO) or 5μM of tazemetostat (Taz). CellTiter-Glo^®^ luminescent cell viability assay was performed after 6 days of treatment. *Columns,* means of values obtained from 4 or 5 independent biological replicates; *white*, vehicle treatment; *red*, tazemetostat treatment; bars, SEM values; –, p-value > 0.05 (not significant); *, p-value < 0.05, all statistical analyses used Wilcoxon tests. **(B)** (top) WCM12, NEPC patient-derived xenograft (PDX) model were grown subcutaneously in SCID mice. Mice were randomized into treatment groups when tumor volume reached 100mm^3^. Mice were treated with vehicle (n=4) or 250 mg/kg b.i.d. of tazmetostat (EPZ, n=4) for 18 days. Two mice in the EPZ were sacrificed before experimental endpoint adhering to animal welfare guidelines. *Connected dots*, means of values obtained from independent biological replicates; *gray*, vehicle treatment; *red*, tazemetostat treatment; bars, SEM values. (bottom) MSKPCa4, NPEC organoid-derived xenograft model were grown subcutaneously in both flanks of the mice and randomized into treatment groups when tumor volume reached approximately 200mm^3^. Mice were treated with vehicle (n=5) or 150mg/kg GSK126 (n=5) for 6 weeks. *Connected dots*, means of values obtained from independent biological replicates; *gray*, vehicle treatment*; magenta*, GSK126 treatment; bars, SEM values. **(C)** Western blot analysis (Top panel – AR-driven PRAD; bottom panel – AR-indifferent NEPC) in a parallel experiment of (A) confirming the downregulation of H3K27me3 upon tazemetostat *treatment in vitro*. Blots were reprobed for total-histone-H3 as a loading control. **(D)** Western blot analysis tumor samples from (B - top) confirming the downregulation of H3K27me3 upon tazemetostat treatment *in vivo*. Blots were reprobed for total-histone-H3 and β-actin as loading controls. **(E)** Immunohistochemistry of tumor samples from (B – bottom) confirming the downregulation of H3K27me3 upon GSK126 treatment in vivo.

To understand the efficacy of EZH2 catalytic inhibition across lineage subtypes of prostate cancer, we performed cell viability and tumor growth experiments upon treatment with tazemetostat in a diverse panel of advanced prostate cancer models. As reported previously (19), AR-positive adenocarcinoma cell lines including LNCaP and LNCaP-abl, responded to 5μM tazemetostat treatment over 6 days (Fig. 1A). However, AR-negative NEPC patient-derived organoid models (WCM154, WCM155, WCM1262, WCM1078) (14) showed modest or no response to 5μM tazemetostat. The modest response to EZH2 inhibition was also confirmed using other EZH2 catalytic inhibitors (valemetostat (24), PF-06821497 (25), and GSK126 (26)) two of which are in clinical trials for prostate cancer (NCT04388852, NCT03460977) **(SFig 1A-C)**. The modest response to EZH2 inhibition was validated *in vivo* using an NEPC patient-derived xenograft (PDX) model (WCM12) where we found that the tumor growth rate was not significantly impacted when treated with tazemetostat compared to vehicle (Fig. 1B). An independent NEPC PDX model, MSKPCa4 also showed modest response to GSK126 (EZH2i) treatment (Fig. 1B). Western blotting and immunohistochemistry confirmed on-target activity of EZH2i with a significant reduction in H3K27me3 after tazemetostat *in vitro* and *in vivo* in both PRAD and NEPC models (Fig.1C–E). RNA-Seq also supported on-target EZH2 inhibitor effects with upregulation of gene sets related to genes bearing H3K27me3 in progenitor cells **(SFig 1D)**. Previous studies using PRAD-to-NEPC transitioning models (i.e.*Pten/Rb1/Tp53*-null mouse model and LNCaP 42D^ENZR^) had reported reversal of lineage re-programming and increased AR expression upon EZH2 inhibition (27, 28). However, in our patient-derived NEPC models, we did not observe changes in lineage genes or expression of AR or its target genes (eg., *NKX3–1*) after EZH2 inhibition **(SFig 1E)**, and gene sets related to prostate development were not significantly enriched after EZH2i therapy **(SFig 1F)**. Overall, these results suggest that EZH2 inhibition may not be as effective in NEPC compared to PRAD, and that EZH2 may have a different function in NEPC beyond reversal to a luminal lineage as reported in plasticity models.

### Lineage-specific dichotomy in the canonical activity of EZH2

**Figure 2:**
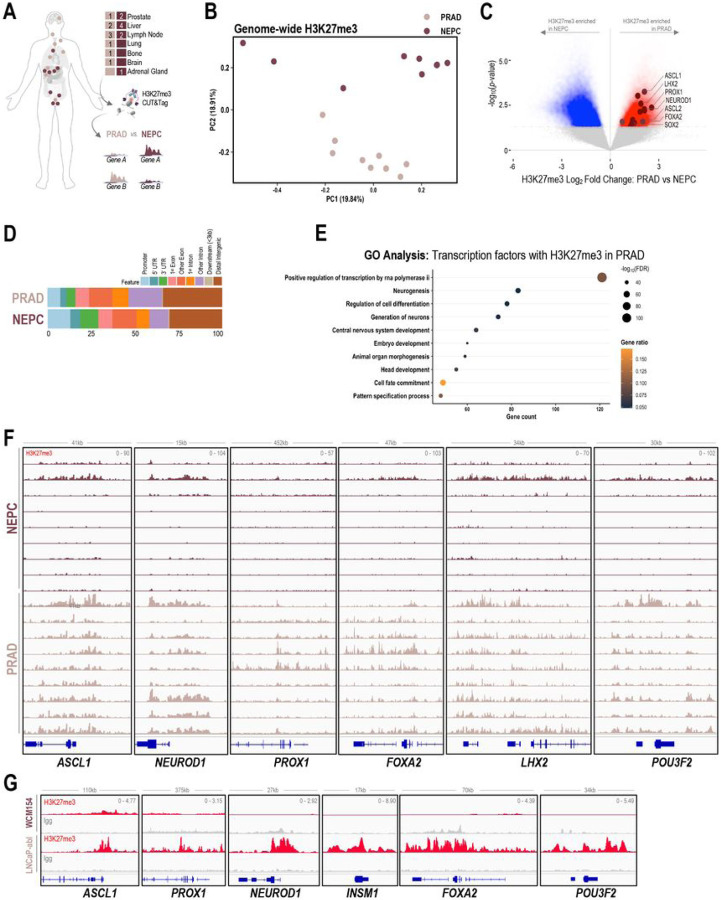
PRC2 targets are distinct in PRAD and NEPC. **(A)** Schematic of rapid autopsy sites of NEPC and castration resistant PRAD and workflow of H3K27me3 CUT&Tag and downstream differential analysis. **(B)** H3K27me3 fragment per million (FPM) reads were calculated genome-wide for 10kb bins for each samples. Principal component analysis of the FPM values from PRAD and NEPC samples across two dimensions; pale pink, castration resistant PRAD; burgundy, NEPC. **(C)** Volcano plot with differentially enriched 10kb bins of H3K27me3 FPM values in NEPC or castration resistant PRAD samples; blue, H3K27me3 regions enriched in NEPC samples; *red,* H3K27me3 regions enriched in castration resistant PRAD samples; *burgundy*, NE-lineage transcription factors with enrichment of H3K27me3 at either promoter or gene body. **(D)** Bar plot showing distribution of subtype specific H3K27me3 enriched in castration resistant PRAD or NEPC; *light blue,* promoter*; blue*, 5’ untranslated regions (UTR); *green*, 3’ UTR; *pale pink*, 1^st^ exon; *coral*, other exon; *orange*, 1^st^ exon; *violet,* other intron; *gray*, downstream (< 3kb); *brown*, distal intergenic. **(E)** Gene ontology analysis of transcription factors with enrichment of H3K27me3 in the promoter of gene body from in castration resistant PRAD (C) with *p-value* < 0.05 and Log_2_FoldChange > 0; *size*, -log_10_FDR; orange-black gradient, gene ratio. **(F)** Screenshots from Integrative Genomics Viewer (IGV) of CUT&Tag H3K27me3 levels in castration resistant PRAD and NEPC and the genomic loci of indicated NE-lineage genes; pale pink, castration resistant PRAD samples; *burgundy*, NEPC samples. **(G)** Screenshots from Integrative Genomics Viewer (IGV) of CUT&RUN H3K27me3 levels in LNCaP-abl and WCM154 and the genomic loci of indicated NE-lineage genes; gray, IgG; red, H3K27me3.

We hypothesized that PRC2 may have different lineage-specific targets in NEPC as compared to PRAD which may contribute to the dichotomy in responses observed in the cell viability and tumor growth studies. To characterize the downstream targets of PRC2, we evaluated the genome-wide distribution of H3K27me3 by CUT&Tag in patient samples collected from rapid autopsy (9 castration-resistant PRAD, 9 NEPC) (Fig. 2A, **STable 1)**. Principal component analysis (PCA) of H3K27me3 signal in genome-wide 10kb bins from patient samples demonstrated a separation of castration-resistant PRAD and NEPC samples along one of the dimensions (PC2) and a modest overlap in the other (PC1) (Fig. 2B). This was validated using UMAP and hierarchical clustering **(SFig 2A-B)**. PCA and UMAP showed similar distinction between PRAD and NEPC samples when analysis was performed using broad H3K27me3 peaks **(SFig 2C)**. Next, we defined 10kb regions of enrichment specific to either PRAD or NEPC and found 30,346 regions specific to PRAD and 46,448 regions specific to NEPC (Fig. 2C). Globally, about 30% of the differentially enriched H3K27me3 regions mapped to distal intergenic regions consistent with previous reports (29), and phenotype-specific distributions were similar between castration-resistant PRAD and NEPC (Fig. 2D). Gene ontology analysis of transcription factors bearing higher levels of H3K27me3 on promoters and gene bodies in PRAD revealed enrichment of neurogenesis and central nervous system development pathways (Fig. 2E). These also included NE lineage transcription factors such as *ASCL1, PROX1, NEUROD1, LHX2, FOXA2, POU3F2*, and *INSM1* harboring high levels of H3K27me3 throughout the genomic loci and/or upstream of transcriptional start sites in PRAD and less enriched, yet present at low levels in NEPC (Fig. 2F, 2C). Transcription factors with high levels of H3K27me3 in NEPC as compared to castration-resistant PRAD included those related to epithelial development based on gene ontology analysis, such as *HOXB13, REST, YAP1, GATA2*. **(SFig 2D-E)**. H3K27me3 at the genomic loci of luminal lineage genes such as *AR, HOXB13*, and *YAP1* was present at higher levels in NEPC compared to PRAD (SFig 2F), however the difference in enrichment was not as dramatic as the NE-lineage genes represented in Fig. 2F. We performed H3K27me3 CUT&RUN in two representative PRAD and NEPC models (LNCaP-abl and WCM154, respectively) which recapitulated the H3K27me3 pro les observed in clinical samples at the loci of the NE and luminal lineage transcription factors (Fig. 2G, **SFig 2G**). These results confirm that PRC2 targets are distinct in castration-resistant PRAD as compared to NEPC, with NE-lineage transcription factors bearing higher levels of H3K27me3 associated with their transcriptional repression in PRAD.

### Bivalent promoters are immediate targets of EZH2 inhibition

**Figure 3:**
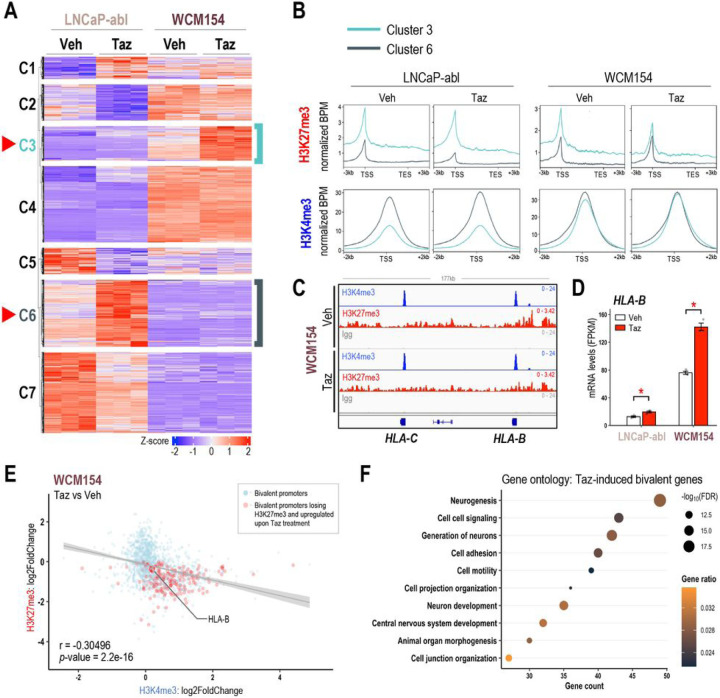
EZH2 inhibition induces bivalent promoters in NEPC. **(A**) Clustering analysis of RNA-seq data from Figure 1E. Unsupervised k-means clustering was performed on scaled (z-score) FPKM values of genes that were upregulated upon tazemetostat treatment in both models (log_2_FC > 0, FDR < 0.05). Optimum number of clusters was determined using sum of squared errors (k=7). Z-score: *red*, high; *blue*, low; *turquoise,* cluster 3 corresponding to genes tazemetostat-induced in WCM154; *teal*, cluster 6 corresponding to genes tazemetostat-induced in LNCaP-abl **(B**) H3K27me3/H3K4me3 CUT&RUN from LNCaP-abl and WCM154 was analyzed and plots show profiles of normalized H3K27me3 and H3K4me3 peaks at ±3kb of gene bodies or TSS of genes upregulated upon EZH2i treatment in LNCaP-abl (*turquoise*) or WCM154 (*teal*). **(C)** Screenshots from Integrative Genomics Viewer (IGV) of CUT&RUN H3K27me3 or H3K4me3 or IgG levels in LNCaP-abl and WCM154 and the genomic loci of indicated MHC class I genes with vehicle or tazemetostat treatment; gray, IgG; red, H3K27me3; blue, H3K4me3. **(D)** FPKM values of HLA-B from the RNA-seq (Figure 1E) in vehicle or tazemetostat treated conditions in indicated models. *Columns*, means of values obtained from 3 independent biological replicates; *white*, vehicle treatment; *red*, tazemetostat treatment; *bars*, SEM values; *, p-value < 0.05. **(E)** Scatter plot of log2FoldChange of H3K27me3 and H3K4me3 upon tazemetostat treatment as described in (B) on bivalent promoters in WCM154; *light blue*, bivalent promoters in vehicle condition; *red*, bivalent promoters losing H3K27me3 and corresponding genes upregulated upon tazemetostat treatment (log_2_FoldChange > 0; *p-value* < 0.05); r value corresponding *p-value* calculated using pair-wise correlation analysis. **(F)** Gene ontology of bivalent promoters losing H3K27me3 and corresponding genes upregulated upon tazemetostat treatment (log_2_FoldChange > 0; p-value < 0.05;) as described in (E); *size*, -log_10_FDR; *orange-black gradient*, gene ratio.

Given the lineage specificity of PRC2 targets, we sought to isolate lineage-specific genes that are induced upon EZH2 inhibition and to understand changes in their epigenetic profiles. We investigated the transcriptomic changes after tazemetostat treatment in LNCaP-abl (castration-resistant PRAD), which had shown the most reduction in cell viability (Fig. 1A) and in WCM154 (NEPC) which showed no response. Unsupervised k-means clustering of RNA-Seq data revealed 7 distinct clusters of genes significantly altered by tazemetostat treatment. Clusters 3 and 6 represented genes that were upregulated in WCM154 and LNCaP-abl respectively in a model-specific manner (Fig. 3A; **SFig 3A**). We confirmed lineage-specificity of tazemetostat-mediated induction of cluster 3 and cluster 6 genes in other PRAD and NEPC models **(SFig 3B)**. As expected, tazemetostat treatment specifically lowered H3K27me3 in cluster 3 and cluster 6 genes in WCM154 and LNCaP-abl, respectively (Fig. 3B). Of note, basal expression of cluster 6 genes was higher than cluster 3 genes in LNCaP-abl and vice versa in WCM154. In LNCaP-abl, the lower expression of cluster 3 genes was associated with higher levels of H3K27me3 in LNCaP-abl (Fig. 3B). However, in WCM154, there was a disconnect between H3K27me3 and mRNA expression of cluster 3 genes, as they harbored higher levels of the repressive H3K27me3 mark and were also highly expressed at baseline (Fig. 3B). This led us to suspect that cluster 3 genes in WCM154 may bear bivalency in their promoters. Bivalent promoters harbor both the transcriptional repressive H3K27me3 and activation H3K4me3 marks and have been reported as immediate targets of PRC2-loss (18). CUT&RUN for H3K4me3 confirmed its presence at high levels in both clusters 3 and 6 in WCM154 (Fig. 3B). MHC class I genes have been reported to have conserved bivalent promoters and specifically highlighted in other small cell neuroendocrine carcinomas (30). We also observed bivalency in MHC class I genes and loss of H3K27me3 upon tazemetostat treatment accompanied by induction in expression (Fig. 3C–D). We validated induction of MHC class I gene, *HLA-B* upon tazemetostat treatment in NEPC models both at the mRNA and protein level (Fig. 3D; **SFig 3C-D**). We evaluated two other genes *TMEFF2* and *SYPL2*, with prominent bivalent profiles at their promoters, and verified loss of H3K27me3 and a corresponding increase in mRNA levels upon EZH2 inhibition **(SFig E-G)**. Of note, these genes harbored bivalency only in WCM154 and not in LNCaP-abl **(SFig H)**. When characterizing the regulation of all bivalent promoters in WCM154 upon EZH2 inhibition, we noted a moderate yet statistically significant inverse correlation in bivalent promoters with gene expression, as tazemetostat led to loss of H3K27me3 and gain of H3K4me3 in WCM154 (Fig. 3E). Interestingly, gene ontology analysis of tazemetostat-induced bivalent promoters with loss of H3K27me3 revealed enrichment for neurogenesis and neuron development pathways (Fig. 3F; **SFig I**). These results indicate that distinct PRC2 targets in PRAD and NEPC contribute to differential gene induction upon tazemetostat treatment, and bivalent promoters may be preferentially induced upon EZH2 inhibition.

### Induction of NE-lineage genes upon EZH2 inhibition contributes to maintenance of a terminally differentiated state

**Figure 4:**
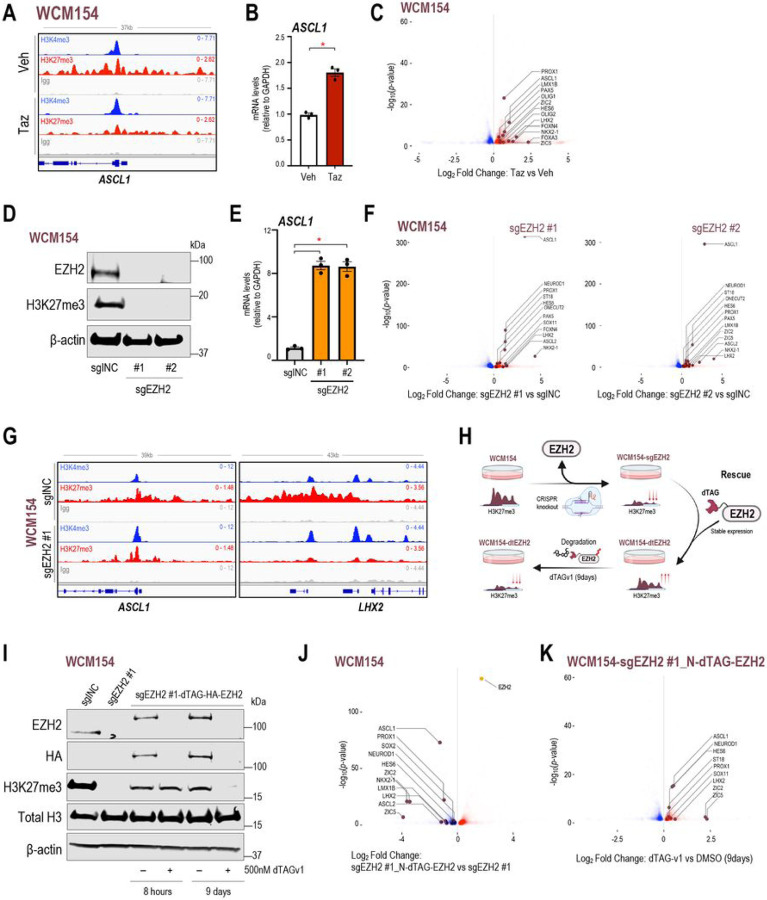
EZH2i in NEPC models induce NE-lineage genes bearing bivalent promoters. **(A)** Screenshots from Integrative Genomics Viewer (IGV) of CUT&RUN H3K27me3 or H3K4me3 or IgG levels in WCM154 treated with vehicle or tazemetostat as described in Figure 1A at the genomic loci of *ASCL1* with vehicle or tazemetostat treatment; *gray*, IgG; *red*, H3K27me3; *blue*, H3K4me3. **(B)** WCM154 was treated as mentioned in Figure 1A and samples were processed for qRT-PCR. Target gene (ASCL1) mRNA levels were normalized to GAPDH expression and are represented as relative expression using one of the values obtained from vehicle-treated conditions as 1*. Columns*, means of values obtained from 3 independent biological replicates; *white*, vehicle; *red*, tazemetostat; *bars*, SEM values; *p-value < 0.05. **(C)** Volcano plot of differentially expressed genes in WCM154 RNA-Seq data as described in Figure 1E; *blue,* significantly downregulated genes upon tazemetostat treatment (log_2_FoldChange < 0; *p-value* < 0.05); *red,* significantly upregulated genes upon tazemetostat treatment (log_2_FoldChange < 0; p-value < 0.05); burgundy, NE-lineage transcription factors upregulated upon tazemetostat treatment. **(D)** Western blot analysis for indicated proteins showing efficiency of knockout of EZH2 in WCM154 using two independent sgRNAs targeting EZH2. Blots were reprobed for β-actin as loading control. **(E)** WCM154 control or EZH2 knockout lines were processed for qRT-PCR. Target gene (*ASCL1*) mRNA levels were normalized to GAPDH expression and are represented as relative expression using one of the values obtained from WCM154-sgINC conditions as 1. *Columns*, means of values obtained from 3 independent biological replicates; *white*, WCM154-sgINC; *orange*, WCM154-sgEZH2 (#1 and #2); *bars*, SEM values; *p-value < 0.05. **(F)** Volcano plot of differentially expressed genes in WCM154 RNA-Seq data as described in (D); *blue*, significantly downregulated genes upon EZH2 knockout treatment (log_2_FoldChange < 0*; p-value* < 0.05); red, significantly upregulated genes upon EZH2 knockout treatment (log_2_FoldChange < 0; *p-value* < 0.05); *burgundy*, NE-lineage transcription factors upregulated upon EZH2 knockout. **(G)** Screenshots from Integrative Genomics Viewer (IGV) of CUT&RUN H3K27me3 or H3K4me3 or IgG levels in WCM154-sgINC or sgEZH2 #1 as described in (D) at the genomic loci of *ASCL1* or *LHX2; gray*, IgG; *red*, H3K27me3; *blue*, H3K4me3. **(H)** Schematic of the WCM154 knockout of endogenous EZH2 and rescue using a recombinant dTAG-fused EZH2 and treatment with degrader compound (dTAGv-1) for 9 days. H3K27me3 levels are lost after EZH2 knockout and rescued (partially) after re-introduction of recombinant dTAG-EZH2 and again lowered upon 9 days of dTAGv-1 mediated degradation of EZH2. **(I)** Western blot analysis for indicated proteins showing efficiency of knockout of EZH2 and rescue using recombinant dTAG-EZH2 in WCM154. Impact of dTAGv-1 treatment at 8 hours and 9 days is shown on levels of EZH2 and H3K27me3. Blots were reprobed for β-actin as loading control. **(J)** WCM154 models with knockout of EZH2 (sgEZH2 #1) and rescue using recombinant dTAG-EZH2 were processed for RNA-Seq. Volcano plot of differentially expressed genes; *blue*, significantly downregulated genes upon EZH2 knockout treatment (log_2_FoldChange < 0; *p-value* < 0.05); *red*, significantly upregulated genes upon EZH2 knockout treatment (log_2_FoldChange < 0; *p-value* < 0.05); *orange*, EZH2 levels shown as control; *burgundy*, NE-lineage transcription factors upregulated upon rescue of EZH2 expression. **(K)** WCM154 models with recombinant dTAG-EZH2 treated with vehicle or dTAGv-1 for 9days were processed for RNA-Seq. Volcano plot of differentially expressed genes; *blue*, significantly downregulated genes upon dTAGv-1 treatment (log_2_FoldChange < 0; *p-value* < 0.05); *red*, significantly upregulated genes upon dTAGv-1 treatment (log_2_FoldChange < 0*; p-value* < 0.05); *burgundy*, NE-lineage transcription factors upregulated upon rescue of EZH2 expression.

The enrichment of pathways related to neurogenesis and neuron development upon tazemetostat treatment in NEPC may justify the lack of lineage reversal and maintenance of terminal differentiation that we observed in patient-derived models of NEPC. *ASCL1*, a well-established NE-lineage driver, also a possessed bivalent promoter in NEPC and was impacted by EZH2 inhibition resulting in its upregulation (Fig. 4A, B). A number of other NE-lineage transcription factors including *PROX1, NKX2–1, OLIG1, OLIG2, ZIC1, ZIC5*, and *FOXN4* were also induced upon tazemetostat treatment in NEPC (Fig. 4C) and exhibited bivalency at their promoters **(SFig 4A-B)**. EZH2 inhibition also led to an increase in expression of NE-lineage transcription factors in other NEPC models WCM155 and WCM1262 **(SFig 4C)**. Of note, several NE-lineage adhesion or secreted proteins, including *DLL3, NPTX1, NRXN2, NTRK2, NRP2* were upregulated upon EZH2 inhibition in NEPC models and portrayed bivalency in their promoters **(SFig 4D-E)** (31, 32).

Using CRISPR-cas9, we established a knockout of EZH2 in WCM154 with two independent guide RNAs which subsequently led to marked reduction in H3K27me3 levels (Fig. 4D). There was an 8-fold increase in *ASCL1* mRNA levels after EZH2 knockout by qRT-PCR analysis (Fig. 4E). RNA-Seq of WCM154 with EZH2 knockout revealed multiple NE-lineage transcription factors including *NEUROD1, PROX1, LHX2, ONECUT2, NKX2–1* as significantly upregulated compared to control WCM154 (Fig. 4F), and this was accompanied by loss of bivalency (Fig. 4G). We rescued EZH2 knockout WCM154 with a recombinant dTAG-HA-EZH2 accompanied with recovery of H3K27me3 levels (Fig. 4H). RNA-Seq comparing WCM154 rescued with dTAG-HA-EZH2 versus WCM154 with EZH2 knockout verified the significant downregulation of NE-lineage transcription factors including *ASCL1, PROX1, NEUROD1, and LHX2* (Fig. 4I). The dTAG system facilitates rapid targeted degradation of EZH2 within minutes after treatment with the heterobifunctional compound dTAGv1 (33). Degradation of EZH2 after 8 hours did not impact its canonical substrate H3K27me3 consistent with previous reports that describe replication dilution of H3K27me3 upon PRC2 loss (Fig. 4J) (34). We observed marked downregulation of H3K27me3 after 9 days of dTAGv1-mediated degradation of EZH2 and RNA-seq once again confirmed the differential upregulation of NE-lineage transcription factors after 9 days of dTAGv1 treatment compared to vehicle control (Fig. 4K). Taken together, these results demonstrate that EZH2 inhibition in terminally differentiated NEPC models leads to a further induction of NE-lineage transcription factors bearing bivalency which may be responsible for maintenance of NE differentiated state.

### Co-activator function of EZH2 may not be prominent in NEPC providing rationale for cyclin-CDK inhibition

**Figure 5:**
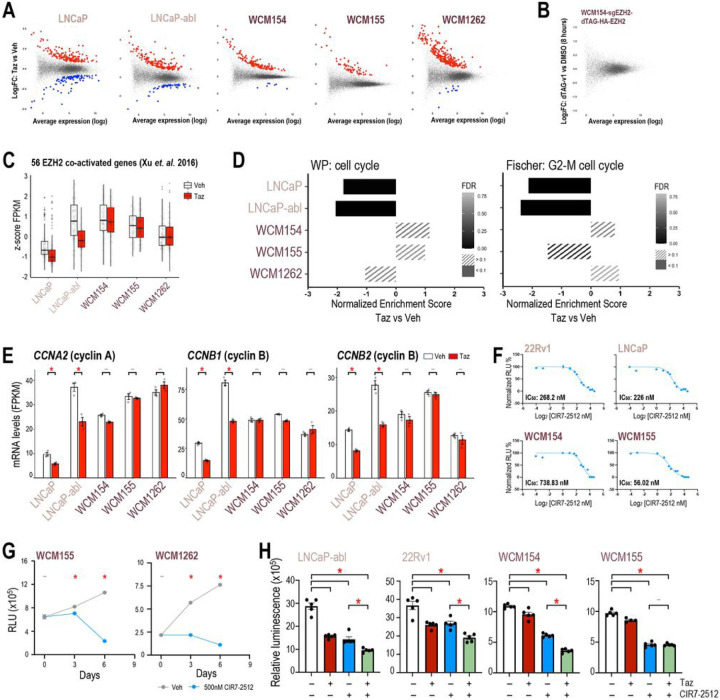
Non-canonical activity of EZH2 in limited to PRAD models. **(A)** Scatter plot of differentially expressed genes in PRAD and NEPC models in RNA-Seq data as described in Figure 1E; *blue*, significantly downregulated genes upon tazemetostat treatment (log_2_FoldChange < 0.5; *p-value* < 0.05); *red*, significantly upregulated genes upon tazemetostat treatment (log_2_FoldChange > 0.5; *p-value* < 0.05). **(B)** Scatter plot of differentially expressed genes in WCM154-sgEZH2 #1-dTAG-N-EZH2rescue model in RNA-Seq data as described in Figure 4H upon treatment with vehicle or dTAGv-1 treatment for 8 hours (log_2_FoldChange < 0.5; *p-value* < 0.05). **(C)** Box plot summary of cumulative z-score of FPKM values of 56 genes co-activated by EZH2 reported in Xu et. al. 2016; *white*, vehicle treatment; *red,* tazemetostat treatment; *bars*, SEM values. **(D)** Cells or organoids of PRAD (*pink*) or NEPC (*burgundy*) models were treated as indicated in Figure 1E as independent biological triplicates and RNA was isolated and processed for RNA-seq. GSEA analysis was performed and normalized enrichment score are plotted for the indicated pathway; *gray-black gradient*, FDR values. **(E)** FPKM values of *CCNA2, CCNB1*, and *CCNB2* from the RNA-seq (Figure 1E) in vehicle or tazemetostat treated conditions in indicated models. *Columns*, means of values obtained from 3 independent biological replicates; *white*, vehicle treatment; *red,* tazemetostat treatment; *bars*, SEM values; –, *p-value* < 0.05; *, *p-value* < 0.05. **(F)** IC50 dose response curves in PRAD (*pale pink*) and NEPC (*burgundy*) using increasing dose of CIR7–2512. *dots*, means of values obtained from 5 independent biological replicates; *bars*, SEM values. **(G)** NEPC (*burgundy*) models were treated with vehicle (Veh; DMSO) or 500nM CIR7–2512 for indicated number of days and cell viability was performed. *dots*, means of values obtained from 5 independent biological replicates; *grey*, vehicle treatment; *blue*, tazemetostat treatment; *bars*, SEM values; –, *p-value* < 0.05; *, *p-value* < 0.05. **(H)** Prostate cancer models (Left panel – AR-driven PRAD; right panel – AR-indifferent NEPC) were treated with vehicle (Veh; DMSO) or 5μM of tazemetostat (Taz) or 500nM CIR7–2512 or in combination. CellTiter-Glo^®^ luminescent cell viability assay was performed after 6 days of treatment. *Columns*, means of values obtained from 4 or 5 independent biological replicates; *white*, vehicle treatment; *red*, tazemetostat treatment; *blue*, CIR7–2512 treatment; *green*, combination treatment; *bars*, SEM values; –, *p-value* > 0.05 (not significant); *, p-value < 0.05, all statistical analyses used Wilcoxon tests.

Based on the differential canonical PRC2-associated function of EZH2 observed in PRAD as compared to NEPC, we posited that the non-canonical activity of EZH2 may also be divergent. We sought to better understand the EZH2 co-activator function and its relevance in the absence of AR signaling in NEPC by looking at genes that are downregulated with tazemetostat. After tazemetostat treatment, 93 and 54 genes were significantly downregulated in LNCaP and LNCaP-abl, respectively, which is approximately one-third of the number of genes upregulated (Fig. 5A). However, in NEPC models only up to 10 genes were significantly downregulated, which suggests less impact of non-canonical EZH2 activity in NEPC (Fig. 5A). To validate this further, we employed our rescue WCM154 model with dTAG-HA-EZH2. dTAG system has been used to identify direct transcriptional targets of transcription factor after short-term degradation and transcriptomic analysis (35–37). RNA-Seq after 8 hours of degradation of EZH2 showed no differentially expressed genes in WCM154 confirming that EZH2 has limited co-activator functions in NEPC (Fig. 5B). A robust gene signature containing 56 genes that represent EZH2-activated genes has been reported in PRAD and validated in hematological malignancies (20). After tazemetostat, we found that the majority of these genes were significantly downregulated in PRAD models but not in NEPC (Fig. 5C; **SFig 5A**). Another recent independent study by Wang *et. al*. (21) of non-canonical EZH2 activating function primarily using the 22Rv1 castration-resistant PRAD model reported 130 EZH2 co-activated genes. We observed a similar pattern of these genes as downregulated in PRAD but not in NEPC models. The non-canonical co-activator function of EZH2 in PRAD mainly impacted E2F gene signature and cell cycle regulation ((19), **SFig 5C, SFig 5D,E)**. Gene-set enrichment analysis pointed to several pathways related to cell cycle regulation that showed high negative enrichment in PRAD but not in NEPC (Fig. 5D). Tazemetostat-mediated downregulation of cell cycle and check point genes including cyclinA and cyclinB was restricted to PRAD, with no notable regulation observed in NEPC despite high basal expression of these genes (Fig. 5E, **SFig 5F**). Gene ontology analyses further confirmed that tazemetostat specifically decreased the expression of genes related to cell cycle processes (cluster2; Fig. 3A) in LNCaP-abl but not in WCM154 **(SFig. 5G)**. Since downregulation of cell cycle regulators upon EZH2 inhibition could contribute to the responses seen with tazemetostat in PRAD, we hypothesized that targeting cell cycle regulators may compensate for the lack of response to EZH2 inhibition in NEPC.

CIR7–2512 is a macrocyclic cyclin-CDK2 inhibitor that blocks RxL substrate binding of the complex (38, 39). We performed IC_50_ dose response analysis of CIR7–2512 for cell viability in two PRAD and two NEPC models along with an inactive enantiomer of the compound (Fig. 5F; **SFig 6A**). CIR7–2512 treatment impeded the growth of WCM155 and WCM1262 over 6 days of treatment (Fig. 5G). Treatment with CIR7–2512 reduced cell viability of PRAD comparable to tazemetostat treatment, and NEPC models that did not respond to tazemetostat treatment showed significant response to cyclin-CDK2 inhibition (Fig. 5H). These effects were validated with another EZH2i (SFig 6B). Strong synergistic effects of combination treatment were not anticipated based on our hypothesis and was not observed in our growth assays (Fig. 5H; **SFig 6B**). Effects of cell-cycle inhibition in PRAD and NEPC models were verified with an independent CDK2 inhibitor, tagtociclib **(SFig 6C**; (40, 41)). Overall, the non-canonical co-activator function of EZH2 may not be prevalent in NEPC and investigating its relevance suggested a strategy for targeting cyclin-CDK2 as an alternative to EZH2 inhibition.

## Discussion

**Figure 6:**
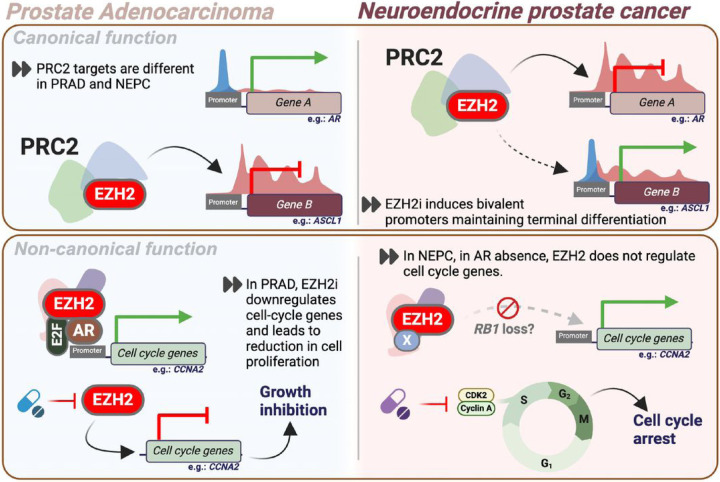
Legend not included with this version.

EZH2 is an emerging therapeutic target in advanced prostate cancer yet clinical biomarkers of response have not been established. While EZH2 is known for its modulation of AR signaling in both AR-driven PRAD and in lineage plasticity models, the function of EZH2 in NEPC has not been defined (19, 21, 27, 28, 42). Based on its important role in differentiation (13), we hypothesized that EZH2 would have variable functions in prostate cancer depending on the underlying differentiation state or phenotype (Fig. 6).

Although EZH2 is overexpressed in nearly all NEPC tumors and has been suggested as a therapeutic target for NEPC, we observed only modest response to EZH2 inhibition in various NEPC preclinical models. EZH2 inhibition in NEPC did not result in lineage reversal back towards PRAD, but surprisingly led to a further induction of the expression of NE-lineage genes potentially pushing tumors towards a more terminally differentiated state. Epigenetic profiling coupled with genetic perturbation identified genes including NE-lineage transcription factors that acquire bivalency in their promoter preparing them to be induced as immediate targets of EZH2 inhibition. Genes such as *ASCL1, NEUROD1, ONECUT2, DLL3*, that are commonly overexpressed in NEPC, acquire H3K4me3 marks of transcriptional activation retaining low levels of H3K27me3 rendering them bivalent and poised for induction after blocking the canonical EZH2 function **(SFig 7)**. While acquired bivalency tissue-specific is a well-studied mechanism of cell differentiation during normal development (17, 18, 43), its parallel in cancer lineage plasticity and drug resistance is not well understood (44). Whether pushing tumors to become more differentiated or neuroendocrine, and less heterogeneous, leads to new or enhanced therapeutic vulnerabilities to NEPC-directed therapies (e.g., DLL3 targeted agents) warrants further study.

MHC class I genes were also identified as bivalent genes upregulated after EZH2 inhibition in NEPC, suggesting an immune modulatory function of PRC2 and pointing to the potential for EZH2 and immunotherapy combination strategies. There are several parallels of these findings with other cancer types. Transcriptional repression of MHC class I genes has been reported in other NE carcinomas including small cell lung cancer, merkel cell carcinoma, and neuroblastoma (45, 46), and transcriptional repression of MHC class I genes may also be acquired as a mechanism of resistance to immunotherapy (47). Epigenetic recovery of MHC class I expression mediated by EZH2 is associated with loss of NE differentiation in SCLC (48). Furthermore, the MHC class I genes have been reported to be silenced in EGFR-mutant lung adenocarcinoma upon lineage transformation to small cell lung cancer (30).

Response to EZH2 inhibition is primarily mediated by its non-canonical activity (19–21). We report cell-cycle genes as non-canonical targets of EZH2 in PRAD beyond AR. The anti-tumor effects of EZH2 inhibition may be through downregulation of cell-cycle related genes in PRAD and other AR-low NEPC-transitioning models (27, 28), potentially collaborating with its impact on AR signaling. However, this was not observed in AR-negative NEPC models and might have contributed to the limited responses to EZH2 inhibition seen in NEPC **(SFig 7)**. Using a novel macrocyclic inhibitor of cyclin/CDK (CIR7–2512) or the CDK2 inhibitor tagtociclib, we demonstrated *in vitro* activity in PRAD that was comparable to EZH2 inhibition. NEPC cells that were insensitive to EZH2 inhibition responded to both CIR7–2512 and tagtociclib. The interplay between EZH2 and other cell cycle targets (e.g., *AURKA, CHEK1, CDKN1A*) has also been reported in other contexts such as breast cancer, melanoma, non-Hodgkin’s lymphoma, T-cell acute lymphoblastic leukemia (49, 50). EZH2 itself is a downstream target of RB-E2F pathway facilitating cell-cycle control (51). Further supporting this interplay, loss of *RB1* is a mechanism of resistance to EZH2 inhibition in rhabdoid sarcoma (52); upon *RB1* silencing, tazemetostat no longer downregulated *CCNA2*. This is also supported by a recent report that utilized a CRISPR screen in rhabdoid sarcoma and merkel cell carcinoma models and found *RB1* or *TP53* loss as a mediator of resistance to EZH2 inhibition (46, 53). *RB1* is commonly lost in NEPC, and these observations are like what we observed in NEPC models (54). Furthermore, *RB1* loss by itself may contribute to the response to cyclin/CDK inhibition independent of the non-canonical function of EZH2 (54).

A number of EZH2 inhibitors are being evaluated in clinical trials for castration-resistant prostate cancer, and all of these trials are biomarker-unselected (NCT04846478, NCT04179864, NCT03460977). PRC2 complex degraders (PROTACs) targeting EED and EZH2 are also under clinical development (55, 56). Identifying the patients most likely to respond will require extensive correlative analyses as well as an improved understanding of the mechanism of action of EZH2 and PRC2 in heterogeneous clinical, genomic, and phenotypic contexts. Biomarker analysis to identify AR-driven, plasticity, and NEPC subtypes, such as with cell free DNA (cfDNA) epigenetic platforms, could help stratify patients and fuel the development of rational subtype-specific combination strategies (57). Serial cfDNA analyses may also be useful to evaluate changes in differentiation state and active histone marks that occur on EZH2 therapy, with downregulation of AR activity expected after EZH2 inhibition in castration-resistant PRAD, upregulation of AR and downregulation of NE programs in mixed/transition tumors, and upregulation of NE programs in NEPC.

## Methods

### Cell culture and reagents

LNCaP, C4–2, 22Rv1, and HEK293FT cells were purchased from American Type Culture Collection (ATCC). LNCaP, C4–2, and 22Rv1 cells were maintained in phenol red-free RPMI1640 medium (Gibco) that is supplemented with 9% fetal bovine serum (FBS) (Gibco) and 1% antibiotic-antimycotic (Gibco). LNCaP-abl (58) was a generous gift from Dr. Myles Brown’s lab at Dana-Farber Cancer Institute and were maintained in phenol red-free RPMI1640 medium (Gibco) that is supplemented with 9% Charcoal-stripped fetal bovine serum (CSS) (Gibco) and 1% antibiotic-antimycotic (Gibco). HEK293FT cells were grown in phenol red containing Dulbecco’s modified Eagle’s medium (high glucose, Life Technologies) that is supplemented with 9% FBS and 1% antibiotic-antimycotic. All cell lines were kept in a 37°C incubator at 5% CO_2_. Cells that were used in transfection studies were seeded in medium without antibiotics. NEPC organoids, including WCM1078, WCM154, WCM155 and WCM1262 used for this study have been described previously (14). The NEPC organoids were grown with growth-factor-reduced Matrigel (Corning). Five droplets of around 50 μl cell suspension/Matrigel mixture was pipetted onto each well of a six-well cell suspension culture plate (Greiner). The six-well plate was placed into a cell culture incubator at 37°C and 5% CO_2_ for 30 min to solidify the droplets before 2 ml prostate-specific culture medium was added to each well. The organoids were grown in prostate-specific culture medium consisting of Advanced DMEM/F12 (Invitrogen) with GlutaMAX (1×, Invitrogen), HEPES buffer (1 M, Invitrogen), 1% antibiotic-antimycotic (Gibco), B27 supplement (Gibco), N-acetylcysteine 1.25 mM (Sigma-Aldrich), mouse recombinant protein EGF 50 ng/ml (Peprotech), human recombinant FGF-10 20 ng/ml (Peprotech), recombinant human FGF-basic 1 ng/ml (Peprotech), A-83–01 500 nM (Tocris), SB202190 10 μM (Sigma-Aldrich), nicotinaminde 10 mM (Sigma-Aldrich), PGE2 1 μM (Tocris), NRG 100 μl/ml (GenScript), Y-27632–2HC1 10 μM (Selleck), Noggin conditioned medium (10%) and R-spondin conditioned medium (5%). Tazemetostat (xcessbio; cat. no. M60122–10S), valemetostat (selleckchem; cat. no. S8926), GSK126 (xcessbio; cat. no. M60071–2S), PF-06821497 (A21388), CIR7–2512 (CirclePharma), CIR7–2724 (CirclePharma), Tagtociclib (sellckchem; cat. no. S9878) were dissolved in DMSO (sigma) for in vitro studies.

### Clinical samples and H3K27me3 CUT&Tag

Tumor specimens were secured adhering to approved protocols by the DFCI and WCM Institutional Review Boards (nos. 19–883 and 1305013903, respectively). Clinical and information and pathology were collected by medical record review. Tumors histology was classified as castration resistant PRAD or NEPC by board certified pathologist. Upon pathological confirmation of a phenotype, punches were made in frozen OCT section and processed for CUT&Tag sequencing at the Centre for Functional Cancer Epigenetics at DFCI. Sequenced reads were mapped to hg38 using bowtie2 version 2.2.5 and visualized in the gures using IGV, ChIPSeeker package in R, and deepTools (59). Broad peak calling was conducted employing MACS2 (60), with a q-value (FDR) < 0.01 set as the threshold for each CUT&Tag dataset. A union set of peaks was generated using BEDTools (61). The read counts mapped to each peak in each sample were computed from BAM les using chromVAR (62). Subsequently, read counts for each peak were normalized through variance stabilizing transformation from DESeq2 (63). PCA and UMAP for Supplementary Fig. 2 were generated using R.

### Western blotting and immunohistochemnistry

Cells were washed twice with ice-cold phosphate-buffered saline (PBS, Gibco) and harvested in whole-cell lysis buffer (110 mM sodium dodecyl sulfate (SDS), 100 mM dithiothreitol, 80 mM Tris-HCl (pH 6.9), 10% glycerol). Cell lysates were boiled at 95°C for 5min. Equal amounts of protein was estimated by Lowry protocol (RCDC Protein Assay, Bio-Rad) and were subjected to NuPAGE Novex gel electrophoresis (Life Technologies). Based on the predicted molecular weight of the protein of interest, Gel electrophoresis was performed on 4–12% or 10% Bis-Tris NuPAGE gels or 3–8% Tris-Acetate NuPAGE gels. Proteins were transferred onto nitrocellulose membranes (NuPAGE) and blocked with 5% milk or bovine serum albumin prepared in tris-buffered saline with 0.1% Tween-20. Primary antibodies against H3K27me3 (cell signaling technology; cat. no. 9733S; 1:2000 dilution) or total-histone-H3 (cell signaling technology; cat. no. 3638S; 1:5000 dilution) or β-actin (cell signaling technology; cat. no. 8457S or 3700S; 1:5000 dilution) or EZH2 (cell signaling technology; cat. no. 5246S; 1:1000 diltuion), or HA-Tag (cell signaling technology; cat. no. 8457S or 3700S; 1:2000 dilution) were used as indicated in the gures. Near Infra-Red Fluorescent detection was performed using LI-COR secondary antibodies matching the host species of the primary antibodies. LI-COR Odyssey M and imaging software were used to visualize the western blots. Blots were stripped with stripping buffer (Restore Plus Western blot, ThermoFisher Scienti c) and reprobed with additional primary antibodies as required.

Immunohistochemistry was performed as described previously (14).

### Cell viability assays

Cell viability was measured at indicated time points after treatment using CellTiter-Glo assay (Promega) in 96-well plate format as per the manufacturer’s protocol.

### Flow Cytometry Analysis

After live/dead staining with Zombie NIR Fixable Viability Kit (cat. 423106; Biolegend) per the manufacturer’s instructions, single-cell suspensions were stained with fuorophore-conjugated primary antibodies (HLA-ABC-BV510, clone W6/30, cat. 311436; Biolegend) in PBS containing 2% v/v FBS. After washing, cells were resuspended in PBS containing 2% FBS and analyzed on a LSRFortessa flow cytometer (Becton Dickinson). Expression levels were compared with isotype control antibody (mouse IgG2a κ-BV510 Isotype Ctrl, clone MOPC-173; cat. 400268; Biolegend). Data analyses were performed using FlowJo software (TreeStar).

### Generation of cells with stable over-expression

*For EZH2 CRISPR knockout*: One million cells were resuspended in 20 μl of electroporation buffer (P3 solution, Lonza) and mixed ribonuclear protein complexes (RNP) of Cas9 protein with bound gRNAs. Assembly of RNPs was performed with 100 μM of tracrRNA (IDT) with 100 μM crRNAs (sgCtrl: AAA AAT GCC AAA AGT TAC CC, sgEZH2 #1: TGC GAC TGA GAC AGC TCA AG, sgEZH2 #2: TAT GAT GGG AAA GTA CAC G) at 1:1 ratio, incubated at 95°C for 5min followed by cooling to room temperature followed by addition of 20 μM Cas9 protein incubated for 15mins at 37°C. The mixtures were transferred to a 16-well Nucleocuvette Strip and nucleofection was performed in a 4D-Nucleofector (Lonza). Following nucleofection, cells were grown in 6-well plate coated with 1% collagen (ThermoFisher). Single cell selection was performed and colonies were selected based on knockout of EZH2 in western blot analysis.

#### For rescue of EZH2

Vector containing EZH2 cDNA was purchased from Addgene (#81742) and site directed mutagenesis (Agilent) was performed to add a stop codon (Fwd 5’−3’, AGA AAG TTG GGC ATC AAG GGA TTT CCA TTT CTC TTT CGA TG; Rev 5’−3’, CAT CGA AAG AGA AAT GGA AAT CCC TTG ATG CCC AAC TTT CT) and remove the start codon (Fwd 5’−3’, TTC TTC CCA GTC TGG CCG CCA ACT TTT TTG TAC A; Rev 5’−3’, TGT ACA AAA AAG TTG GCG GCC AGA CTG GGA AGA A). Gateway cloning (NEB) LR reaction was performed using the modi ed construct and destination vector (Addgene #91797) to produce pLEX_305-N-dTAG-HAEZH2. The destination vector was a generous gift from Dr. William Kaelin lab. This plasmid was co-transfected with pMD2.G and psPAX2 using Lipofectamine 3000 (Invitrogen) into HEK293FT cells to generate viral particles. Lentiviral supernatants and 10 μg/ml Polybrene were added and incubated with cell lines mentioned in the figures. Infected cell lines were selected with antibiotics as described in the constructs. Over-expression of the desired proteins was confirmed by western blot analysis as described above.

### Epigenetic and transcriptomic studies

#### ChIP-qPCR

ChIP was performed using 5–7 million cells in a 10cm dish. Cells were washed with ice-cold PBS containing EDTA-free protease inhibitor (Roche) and 5mM sodium butyrate. Crosslinking was performed using 1% methanol-free formaldehyde (invitrogen) for 5 mins, quenched by adding 0.125M glycine for 5 mins. Cells were scraped and centrifuged to pellet down at 2000rpm for 2 mins. Pelleted cells were lysed using sarkosyl lysis buffer (0.1%SDS, 1x TX-100, 10mM Tris-HCl pH = 8, 1 mM EDTA pH = 8, 0.1% Sodium Deoxycholate, 0.25% sarkosyl, 0.3 mM NaCl, EDTA-free protease inhibitor (Roche) and 5mM Sodium butyrate). Sonication was performed using Covaris machine in 1ml tubes (Covaris) at 5% duty factor, 140 PIP, 200 cycles per burst, for 5 mins. Chromatin immunoprecipitation was performed overnight using antibodies conjugated with dynabeads (invitrogen) against IgG control (cell signaling technology, cat. no. 3900S, 1 μg/reaction) or H3K27me3 (cell signaling technology, cat. no. 9733S, 1 μg/reaction) or H3K4me3 (cell signaling technology, cat. no. 9751S, 1 μg/reaction). After 5 washes with wash buffer (HEPES-based RIPA buffer), elution buffer (1%SDS, 0.1M NaHCO_3_) was added to the beads. RNAse treatment was performed for an hour at 37°C followed by 16 hours of Proteinase K treatment at 65°C overnight. DNA was purified (Qiagen) and qPCR was performed using primers mentioned in the table below along with 0.1% input.

#### CUT&RUN

CUT&RUN studies were performed with 100,000 cells in duplicates using a kit from Epicypher adhering to manufacturer’s protocol using antibodies against IgG or H3K4me3 (Epicypher, cat. no. 13–0041, 0.5 μg per reaction) or H3K27me3 (Invitrogen, cat. no. MA511198, 0.5 μg per reaction). Automated library-preparation and sequencing was performed by Molecular Biology Core Facilities in Dana-Farber Cancer Institute. Sequencing reads were mapped to hg38 human genome reference using bwa and visualized using deepTools as plotProfiles.

#### RNA-Seq and qRT-PCR

Total RNA isolated (QIAGEN) was submitted to Novogene for paired-end sequencing. Sequencing reads were mapped to hg38 human genome reference using STAR-RSEM. Differential gene expression analysis was performed using the limma and edgeR and GSEA software was used to perform pathway analysis. Gene ontology analysis was performed using hypergeometric overlap statistic tool to calculate the overlap between a gene list and pathways in Molecular signature database. Bar graphs and heatmaps were generated using sample FPKM values.

#### qRT-PCR

RNA was isolated in biological triplicates (QIAGEN) and complementary DNA (cDNA) was prepared from 500 ng total RNA (Bio-Rad). Quantitative Real-time reverse transcription–PCR (qRT–PCR) was done using SYBR Green PCR mastermix (Bio-Rad) on a Bio-Rad qPCR system. Primers sequences are mentioned in table below. Relative quantification was calculated using the △Ct method normalized to GAPDH levels.

**Table:** 

Target	Assay	Forward primer(5’ to 3’)	Reverse primer(5 ‘to 3’)
GAPDH	qRT-PCR	GAAGGTGAAGGTCGGAGTC	GAAGATGGTGATGGGATTTC
ASCL1	qRT-PCR	GAAGATGGTGATGGGATTTC	CAAAGCCCAGGTTGACCA
SYPL2	qRT-PCR	GCTGGGCTTCATCAAAGTTC	GAAGGGATAGCCAAATGCAA
TMEFF2	qRT-PCR	CTGCATGCAAACAGCAGAGT	CTGCACCAAACTGGCAAATA
SYPL2	ChlP-qPCR	CTTAGTGGCAGGAGGGTGAA	ACCAAGGAGTGGTATGTGCAG
TMEFF2	ChlP-qPCR	GGCGTTTGGCAGTCACTTA	GTCATGGTGCTGTGGGAGT

### Data and code availability

All sequencing data have been deposited in the public repository (Gene Expression Omnibus) and code used to generate figures are available upon request.

### Animal studies

All animal studies were approved by the Dana-Farber Cancer Institute (DFCI) or Weill Cornell Medicine Institutional Animal Care and Use Committee (IACUC).

WCM12 PDX tumors were subcutaneously implanted were injected to right fank of 6–8 weeks-old NSG male mice (NOD.Cg-Prkdc^scid^Il2rg^tm1Wjl^/SzJ, the Jackson laboratory). Tumor volumes defined as 0.5 ×.length × width^2^ and the body weights were measured every 3 days. Mice were randomly assigned into vehicle or tazemetostat group once their tumors reach 100mm^3^. Mice were treated with vehicle (0.5% Methylcellulose (400 cps), 0.1% Tween80, pH4.0) or tazemetostat (250 mg/kg) b.i.d. p.o. for indicated number of days. The mice were sacrificed in a CO_2_ chamber adhering IACUC guidelines. At necropsy, tumor size, tumor weight, and body weight were measured to evaluate the drug effect and toxicity.

MSKPCa4 organoids were dissociated into single cells and 4 million cells were mixed 50:50 with Matrigel and injected into CB17 SCID mice for subcutaneous growth. Once the tumors were obtained, they were subsequently passaged *in vivo* for the experiment. GSK126 (150mg/kg) or vehicle was administered q.d. i.p. at a dose volume in 20% captisol adjusted to pH 4–4.5 with 1 N acetic acid.

### Statistical analysis

Standard two-tailed Student t-tests and nonparametric tests were performed to analyze statistical significance of two groups. Two-way ANOVA tests were performed to examine the statistical significance of datasets with grouped analyses. Statistical significance was set at p < 0.05 in this study. All graphs and statistical analyses were completed using GraphPad Prism or ggplot package in R or deepTools. Pearson correlation was used for nearest neighbor analysis and pairwise-correlation.
